# Widespread aggregation of mutant VAPB associated with ALS does not cause motor neuron degeneration or modulate mutant SOD1 aggregation and toxicity in mice

**DOI:** 10.1186/1750-1326-8-1

**Published:** 2013-01-03

**Authors:** Linghua Qiu, Tao Qiao, Melissa Beers, Weijia Tan, Hongyan Wang, Bin Yang, Zuoshang Xu

**Affiliations:** 1 Department of Biochemistry and Molecular Pharmacology, University of Massachusetts Medical School, Worcester, MA, 01602, USA; 2 Current address: Department of Biological Sciences, Wellesley College, Wellesley, MA, 02481, USA; 3 Current address: Division of Biology, California Institute of Technology, Pasadena, CA, 91125, USA; 4 Department of Cell Biology, University of Massachusetts Medical School, Worcester, MA, 01602, USA; 5 Neuroscience Program, University of Massachusetts Medical School, Worcester, MA, 01602, USA

**Keywords:** VAPB, ALS, Motor neuron disease, Neurodegeneration, Transgenic mice

## Abstract

**Background:**

A proline-to-serine substitution at position-56 (P56S) of vesicle-associated membrane protein-associated protein B (VAPB) causes a form of dominantly inherited motor neuron disease (MND), including typical and atypical amyotrophic lateral sclerosis (ALS) and a mild late-onset spinal muscular atrophy (SMA). VAPB is an integral endoplasmic reticulum (ER) protein and has been implicated in various cellular processes, including ER stress, the unfolded protein response (UPR) and Ca^2+^ homeostasis. However, it is unclear how the P56S mutation leads to neurodegeneration and muscle atrophy in patients. The formation of abnormal VAPB-positive inclusions by mutant VAPB suggests a possible toxic gain of function as an underlying mechanism. Furthermore, the amount of VAPB protein is reported to be reduced in sporadic ALS patients and mutant SOD1G93A mice, leading to the hypothesis that wild type VAPB plays a role in the pathogenesis of ALS without VAPB mutations.

**Results:**

To investigate the pathogenic mechanism *in vivo*, we generated human wild type (wtVAPB) and mutant VAPB (muVAPB) transgenic mice that expressed the transgenes broadly in the CNS. We observed robust VAPB-positive aggregates in the spinal cord of muVAPB transgenic mice. However, we failed to find an impairment of motor function and motor neuron degeneration. We also did not detect any change in the endogenous VAPB level or evidence for induction of the unfolded protein response (UPR) and coaggregation of VAPA with muVAPB. Furthermore, we crossed these VAPB transgenic mice with mice that express mutant SOD1G93A and develop motor neuron degeneration. Overexpression of neither wtVAPB nor muVAPB modulated the protein aggregation and disease progression in the SOD1G93A mice.

**Conclusion:**

Overexpression of VAPBP56S mutant to approximately two-fold of the endogenous VAPB in mouse spinal cord produced abundant VAPB aggregates but was not sufficient to cause motor dysfunction or motor neuron degeneration. Furthermore, overexpression of either muVAPB or wtVAPB does not modulate the course of ALS in SOD1G93A mice. These results suggest that changes in wild type VAPB do not play a significant role in ALS cases that are not caused by VAPB mutations. Furthermore, these results suggest that muVAPB aggregates are innocuous and do not cause motor neuron degeneration by a gain-of-toxicity, and therefore, a loss of function may be the underlying mechanism.

## Background

Motor neuron diseases (MND) are a group of diverse neurological disorders with motor neuron involvement that include amyotrophic lateral sclerosis (ALS), primary lateral sclerosis, spastic paraplegias, progressive muscular atrophy, spinal muscular atrophy (SMA), and spinobulbar muscular atrophy [1]. ALS, also referred to as Lou Gehrig’s disease, is the most common adult-onset motor neuron disease caused by degeneration of upper and lower motor neurons, accompanied by progressive weakness, muscle wasting and fasciculations, spasticity, dysarthria, dysphagia, and respiratory compromise. While 90% of ALS cases are sporadic without known genetic mutations, 10% of the cases are familial, which are caused by more than a dozen genetic mutations [2]. Among them, a proline-to-serine substitution at position-56 (P56S) of vesicle-associated membrane protein-associated protein B (VAPB) in the highly conserved major sperm protein (MSP) domain causes some dominantly inherited forms of MND (ALS8), which show typical and atypical ALS symptoms or a mild late-onset spinal muscular atrophy (SMA) [3,4]. A second missense mutation in VAPB causing an amino acid change from threonine to isoleucine at codon 46 (T46I), also in the MSP domain, has been proposed as a causative factor in a single case of familial ALS (fALS) [5].

VAPB belongs to a well conserved VAP family of proteins. VAPB is an integral membrane protein of the endoplasmic reticulum (ER) with an amino terminal MSP domain, a central coiled-coil motif and a carboxy-terminal transmembrane domain anchored in ER membrane [6-12]. VAPB has roles in neurotransmitter release [13], ER to Golgi transport [14,15], bouton formation at the neuromuscular junction [16], Ca^2+^ homeostasis [6,17] and signaling via Eph receptors [18] and Robo and Lar-like receptors [19]. VAPB has also been implicated in other cellular processes, including endoplasmic reticulum (ER) stress and the unfolded protein response (UPR), in which VAPBP56S is functionally abnormal in these processes [5,10,18,20-22]. While some studies have shown that VAPBP56S can enhance ER stress and the UPR [18,21], others have associated VAPBP56S with an inhibitory effect on the UPR [5,10,20,22]. It is unclear what functional and structural changes induced by ALS/MND-related mutations in VAPB lead to neurodegeneration and muscle atrophy in patients.

Similar to many neurodegenerative disorders, the two known VAPB mutants can form aggregates [4,5,7,12,21,23,24], which also sequester wild type VAPB and VAPA [12,25]. It is possible that VAPB mutations cause ALS/MND by a dominant-negative effect and/or a gain of toxicity from its protein aggregates and recruitment of wild type VAPB and VAPA proteins into these aggregates, especially in motor neurons. To test this hypothesis, we generated both wild type VAPB (wtVAPB) and VAPB P56S (muVAPB) transgenic mice with broad expression in the CNS and peripheral tissues and observed robust formation of VAPB positive aggregates in the spinal cord of the muVAPB, but not the wtVAPB transgenic mice. However, we detected neither motor dysfunction nor motor neuron degeneration. These results do not support the gain-of-toxicity mechanism for mutant VAPB-induced ALS.

It has been suggested that VAPB deficiency may play a broad role in the pathogenesis of familial ALS (fALS) and sporadic ALS (sALS) because decreased levels of VAPB have been reported in patients with sALS and the SOD1G93A mouse model [12,26]. On the other hand, VAPBP56S may cause pathogenesis of the disease by a gain of toxic function [27]. Therefore, we crossed both muVAPB and wtVAPB transgenic mice with SOD1G93A mice to determine whether the muVAPB and wtVAPB modulate the course of the disease caused by the SOD1 mutation. If there was a gain of toxic function or a dominant negative effect due to muVAPB overexpression, we expected to observe an adverse effect of muVAPB on SOD1G93A mice, such as accelerated disease onset and progression. Conversely, if the decreased level of VAPB played a significant role in the pathogenesis of ALS, we would observe a slowing in the disease progression in the SOD1G93A and wtVAPB double transgenic mice. However, neither muVAPB nor wtVAPB overexpression affected the disease onset or progression caused by mutant SOD1, suggesting that mutant VAPB does not have a gain of toxicity and VAPB levels do not modulate the disease course of ALS cases without the VAPB mutations.

## Results

### Overexpression of human mutant or wild type VAPB in the transgenic mice

**Figure 1:**
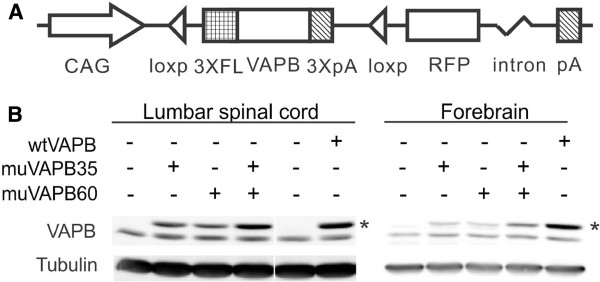
**Overexpression of wtVAPB or muVAPB in the spinal cord and brain of the transgenic mice.** (**A**) Schematic diagram showing the transgene construct. This construct has a CAG promoter, which drives the expression of human VAPB gene that is tagged by 3XFLAG (marked as 3XFL) at its N-terminal. The 3X poly A sequences (3XpA) after the VAPB gene terminate the transcription. Therefore, the RFP gene is not expressed. The two triangles represent the loxP sequence that flank the VAPB transgene. (**B**) VAPB levels from two lines of muVAPB and one line of wtVAPB transgenic mice were determined by Western blot. For the detection of VAPB in the spinal cords, 100 μg protein was loaded in each lane. The proteins were resolved by a 12% SDS-PAGE and detected as described in the materials and methods. For the detection of VAPB in the brains, 30 μg protein was loaded in each lane. Due to its 3XFLAG tag, the molecular weight of the transgenic VAPB (as indicated by *) is slightly more than the endogenous mouse VAPB protein.

To investigate the role of VAPB in ALS, we created both wtVAPB and muVAPB transgenic mice. To differentiate the human VAPB versus the mouse endogenous VAPB, we added a 3xFLAG-tag at the N-terminus of human VAPB. We used a strong and ubiquitously active CAG promoter to drive the expression of the transgenes (Figure 1A). We designed the vectors with the loxP sequences flanking the VAPB transgene, so that if neurodegeneration occurred, we would cross these transgenic mice with various neuron- or glia-specific Cre driver lines to determine the cellular origin of the mutant toxicity, as had been shown in other studies [28,29]. However, we did not carry out these crosses because, as will be detailed below, our transgenic mice did not develop neurodegeneration. Nevertheless, we note that this Cre-loxP approach also carries a technical artifact and the results from such crosses would be difficult to interpret even if neurodegeneration should occur in these mice [30].

The construct was verified by expression of the transgene after transient transfection into HEK293 cells and subsequent Western blot for the FLAG-tagged VAPB protein (data not shown). After pronuclear injection, a total of 14 wtVAPB and 23 muVAPB transgenic founder lines were generated and then screened for the expression of the transgene in brains and spinal cords by Western blot with anti-FLAG or anti-VAPB antibody. One wtVAPB line (wtVAPB) and two muVAPB lines (muVAPB35 and muVAPB60) that showed the highest levels of expression in the spinal cords were further studied. In wtVAPB transgenic mice, the transgenic hVAPB protein level was 2–3 times higher than the mouse endogenous VAPB level in both the spinal cord and forebrain, while the protein levels of muVAPB in the lines muVAPB35 and muVAPB60 were slightly higher than the endogenous mouse VAPB level in the spinal cord and lower than the endogenous level in the forebrain (Figure 1B). To achieve a higher expression level of muVAPB, the two muVAPB lines were crossed to obtain double transgenic mice (muVAPB35/60), which expressed the muVAPB at a level approximately the same as the wtVAPB mice in the spinal cord and approximately equal to the endogenous VAPB level in the forebrain (Figure 1B). Notably, the expression of transgenic VAPB did not alter the expression level of the endogenous VAPB as compared with the level in the non-transgenic (NTG) control. In addition to the CNS, the transgene was also expressed in peripheral tissues, including the skeletal muscle, heart, spleen, liver and lung (data not shown).

### Overexpression of muVAPB, but not wtVAPB, led to VAPB aggregation

**Figure 2:**
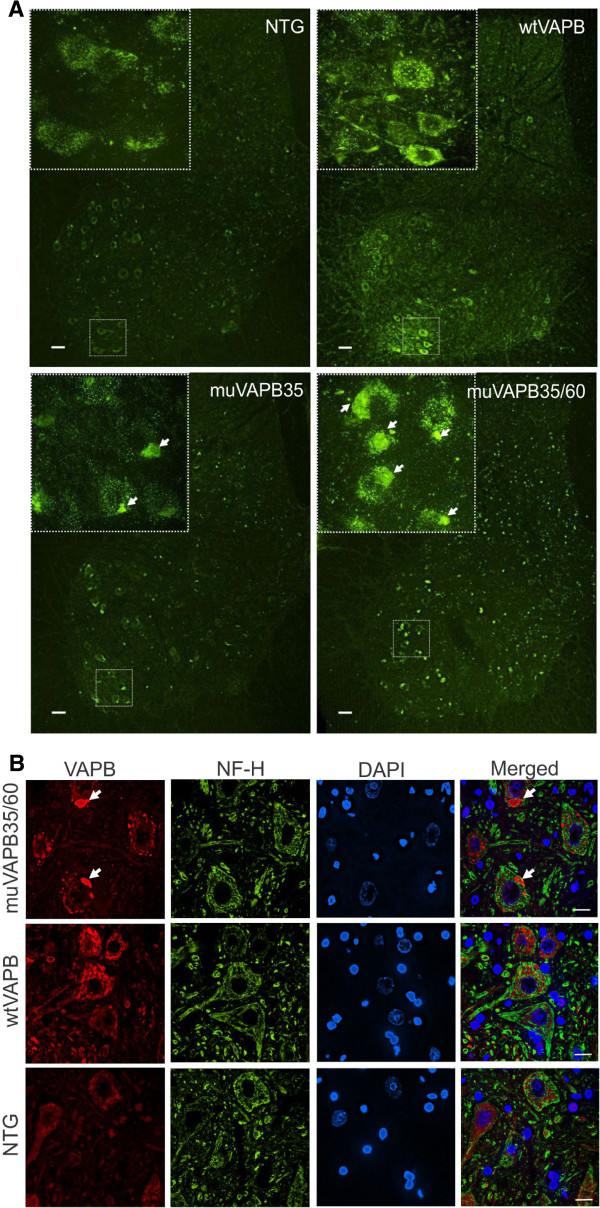
**Overexpression of muVAPB, but not wtVAPB, causes aggregation.** (**A**) Lumbar spinal cord sections from different transgenic lines (NTG, wtVAPB, muVAPB35, and muVAPB35/60) were stained with VAPB antibody. VAPB-positive inclusions were found throughout the whole spinal cord in the muVAPB mice but not in the wtVAPB mice. The inserts show high magnification of the boxed areas in the large image. Aggregates are indicated by arrows. Scale bars: 30 μm. (**B**) Motor neurons in muVAPB mice, but not in wtVAPB mice, show VAPB aggregates. The neurons were marked by staining for neurofilaments high molecular weight subunit (NF-H). The VAPB aggregates are indicated by arrows in the figure. Scale bars: 10 μm.

We examined the expression pattern of the transgene in different cell types in spinal cord sections by immunofluorescence staining. An increased staining intensity was observed in the wtVAPB transgenic lines (Figure 2A,B) compared with NTG mice (Figure 2A,B) but no aggregates were detected. In contrast, muVAPB transgenic mice showed aggregates throughout the whole spinal cord (Figure 2A,B). In the ventral horn area, where motor neurons are located, the aggregates were found in the cytoplasm of the motor neurons in the muVAPB transgenic mice (Figure 2A,B). The aggregates were detected in both mutant lines at similar levels and were increased in number and size in the double transgenic mice gene-rated from crossing the two mutant lines (Figure 2A). Thus, only muVAPB proteins, but not wtVAPB proteins, form aggregates in the transgenic mice.

### muVAPB overexpression did not cause motor dysfunction or affect motor neuron survival

**Figure 3:**
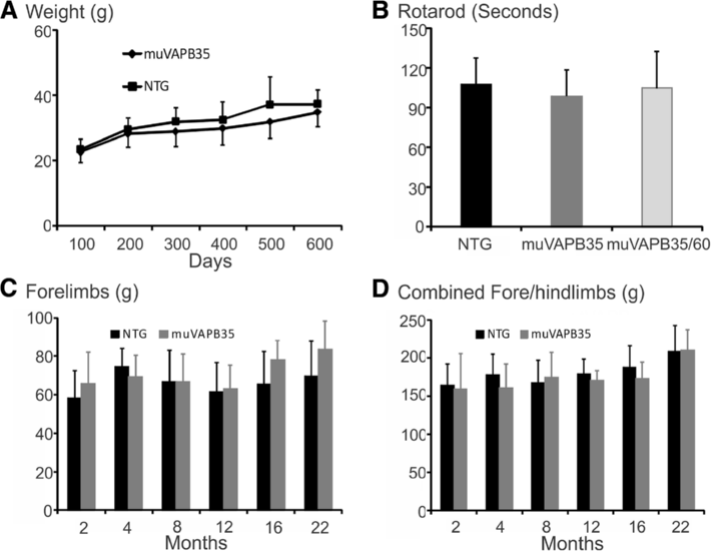
**Age-associated changes in body weight and motor function in VAPB transgenic mice.** (**A**) No significant difference in the body weight between female muVAPB and NTG mice up to 600 days old. For each time point, 6 to 21 mice from each genotype were analyzed. (**B**) No significant difference among muVAPB35/60 (n = 4), muVAPB35 (n = 11) and NTG (n = 11) mice in rotarod test at the age of 10 to 12 months. (**C**, **D**) Grip strength tests of the forelimbs (**C**) and combined forelimbs and hindlimbs (**D**) in female NTG and muVAPB35 transgenic mice at different ages. The number of mice tested from each genotype at each time point is in the range of 4 to 22. All values are displayed as averages with standard deviation.

To determine whether there was any phenotypic consequence of muVAPB expression, we closely monitored any overt phenotypic changes in the muVAPB transgenic mice and did not find any difference from the NTG mice up to >600 days of age. There was no significant difference in body weight between the muVAPB mice and NTG mice as they aged (Figure 3A). To determine whether the motor function was affected, we performed several motor behavioral analyses. We did not detect a significant difference in rotarod performance or grip strength tests between the muVAPB and NTG mice (Figure 3B-D).

**Figure 4:**
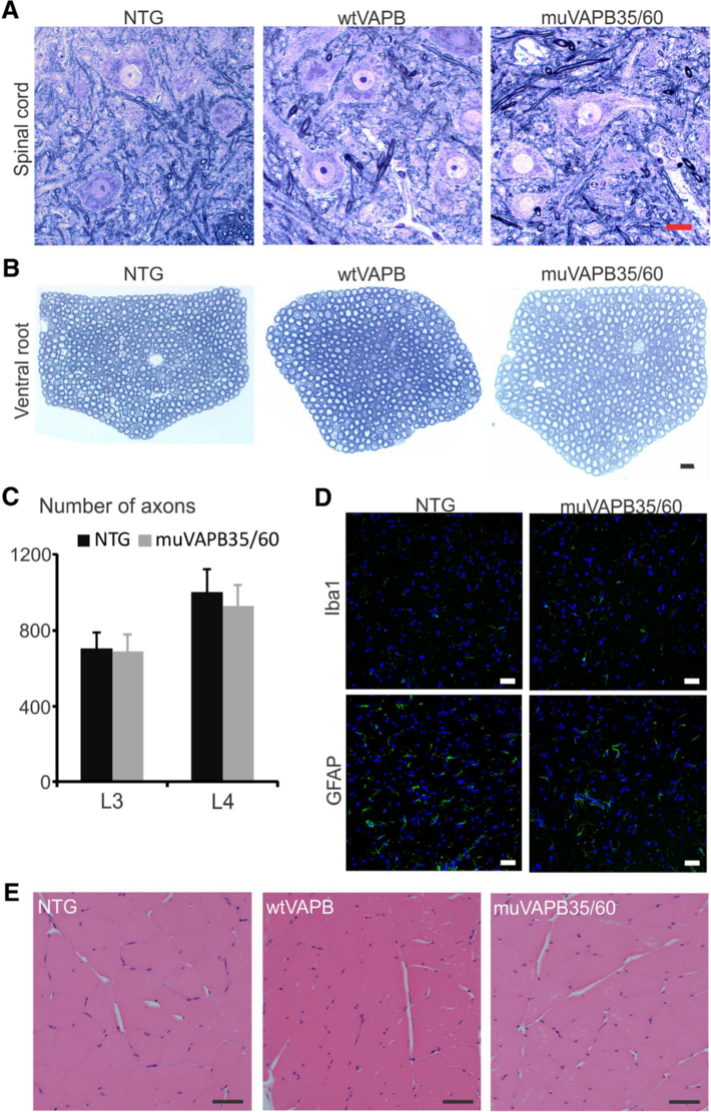
**No significant pathological change in 18-month old VAPB transgenic mice.** Spinal cord sections (**A**) of the ventral horn area and L3 ventral root nerve sections (**B**) from different genotypes are shown. All plastic sections were cut at 1 μm thickness and stained with toluidine blue. Scale bars: 20 μm. (**C**) Numbers of total axons of the ventral roots (L3 and L4) from 18-month old mice were counted. No significant difference in the numbers of axons was found between muVAPB35 (n = 5) and NTG (n = 4) mice. All values are shown as averages with standard deviation. (**D**) No evidence of microgliosis and astrogliosis was detected in the spinal cord of muVAPB35/60 mice, as compared with NTG mice. The sections from the lumbar spinal cords were stained with Iba1 and GFAP antibodies (green fluorescence) to show microglia and astrocytes, respectively. The nuclei were shown by DAPI stain. Scale bars: 20 μm. (**E**) No pathological change in the muscles from transgenic mice. Gastrocnemius muscle sections from NTG, wtVAPB, and mutVAPB35/60 were stained with H&E. Scale bars: 50 μm.

To determine whether there was motor neuron degeneration as a consequence of VAPB aggregation, we examined spinal cords and ventral roots. We detected no evidence for degeneration of motor neurons or their axons in either wtVAPB or muVAPB transgenic mice (Figure 4A,B). Quantification of the total number of ventral root axons from 18-month old muVAPB35/60 mice showed no significant difference from the NTG controls (Figure 4C). Additionally, we detected no evidence for astrogliosis or microgliosis (Figure 4D) and there was no change in muscle morphology indicative of denervation atrophy (Figure 4E). Notably, no evidence of neurodegeneration was found even in the muVAPB35/60 double transgenic mice, where muVAPB expression and aggregation were further elevated (Figures 1, 2 and 4). These observations demonstrate that a 2-fold overexpression of either wtVAPB or muVAPB does not cause motor dysfunction and motor neuron degeneration in mice.

### VAPB aggregation did not lead to typical ALS pathology

**Figure 5:**
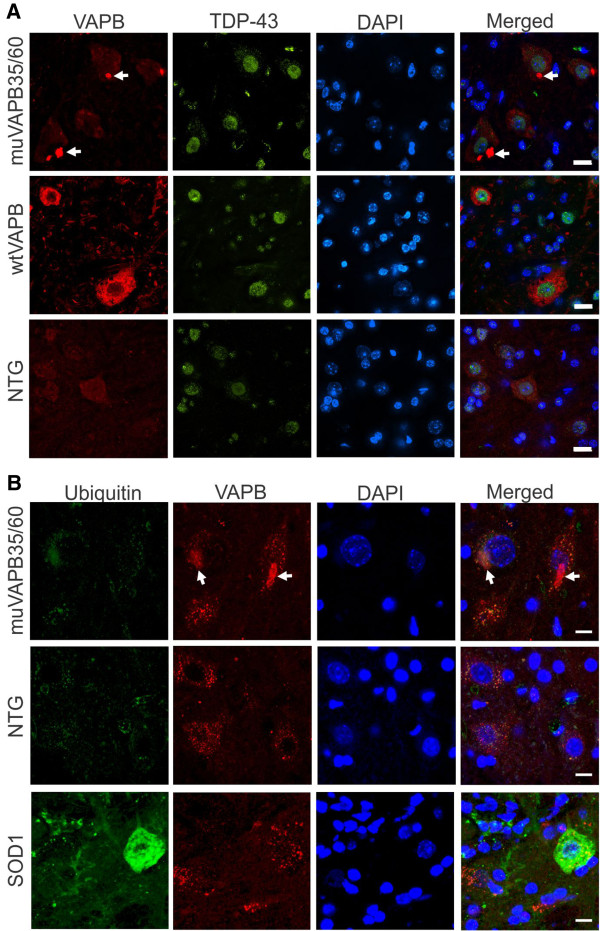
**No significant TDP-43 pathology and ubiquitin-positivity in the muVAPB transgenic mice.** (**A**) No TDP-43 aggregation or change in its cellular distribution in the spinal cords of VAPB transgenic mice was detected. Frozen sections from lumbar spinal cords from NTG, wtVAPB and muVAPB35/60 mice at 18-month old were stained with VAPB and TDP-43 antibodies. Scale bars: 10 μm. (**B**) Ubiquitin staining was absent or weak in motor neurons with muVAPB aggregates. Frozen sections from cervical spinal cords from NTG and muVAPB35/60 at 18-month old were stained with ubiquitin and VAPB antibodies. The sections from mutant SOD1 mice at their end stage were stained in parallel as positive controls. Scale bars: 6 μm. The VAPB aggregates are indicated by arrows in the figure. The nuclei are shown by DAPI stain.

Formation of TAR-DNA binding protein (TARDBP, also known as TDP43)-positive intracellular inclusions in the central nervous system (CNS) is the most common pathology in the patients with ALS and frontotemporal lobar degeneration (FTD), even in the absence of TDP-43 mutations [31-33]. The formation of intracellular TDP-43-containing inclusions is accompanied by a nuclear clearance of TDP-43 [33,34]. However, no information is available regarding the involvement of TDP-43 pathology in patients with VAPB mutations, although a subtle change in TDP-43 distribution and ubiquination have been reported in a VAPBP56S transgenic mouse line [24]. To investigate the correlation between VAPB mutation and TDP-43 pathology, we performed TDP-43 staining in our muVAPB35/60 double transgenic mice, which contain most robust VAPB aggregates. As shown in Figure 5A, we detected no obvious change in TDP-43 staining pattern in the spinal cord motor neurons of 18-month old muVAPB35/60 and wtVAPB transgenic mice.

Protein aggregates in neurodegenerative diseases are marked by ubiquitin and p62, which implies an ongoing clearance process of these aggregates by the cellular protein quality control system [35]. To examine whether there is a clearance process against the VAPB aggregates, we stained motor neurons for ubiquitin and p62. We detected no labeling of ubiquitin in the majority of VAPB aggregates and only occasional weak labeling in some aggregates (Figure 5B, arrows). We also detected no p62 labeling of the VAPB aggregates (data not shown). These results suggest that VAPB aggregates differ from the pathogenic aggregates such as those derived from mutant SOD1 (Figure 5B) that trigger the response from the cellular protein quality control system.

### muVAPB did not cause structural change in ER or coaggregate with VAPA

**Figure 6:**
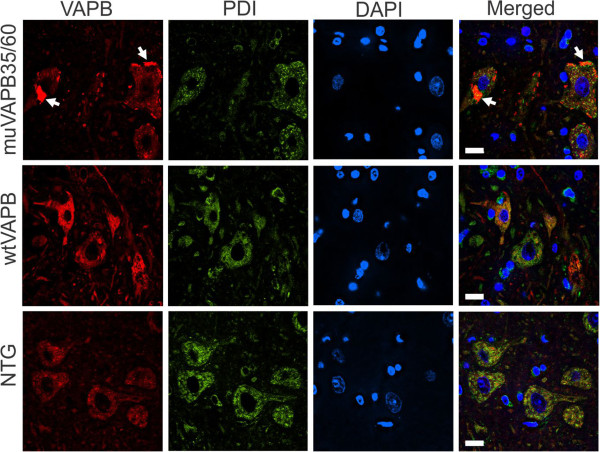
**muVAPB did not affect ER integrity.** Frozen sections from the lumbar spinal cords of NTG, wtVAPB and muVAPB35/60 mice at 18-month old were stained with VAPB and PDI antibodies. VAPB aggregates are indicated by arrows. Scale bars: 10 μm.

**Figure 7:**
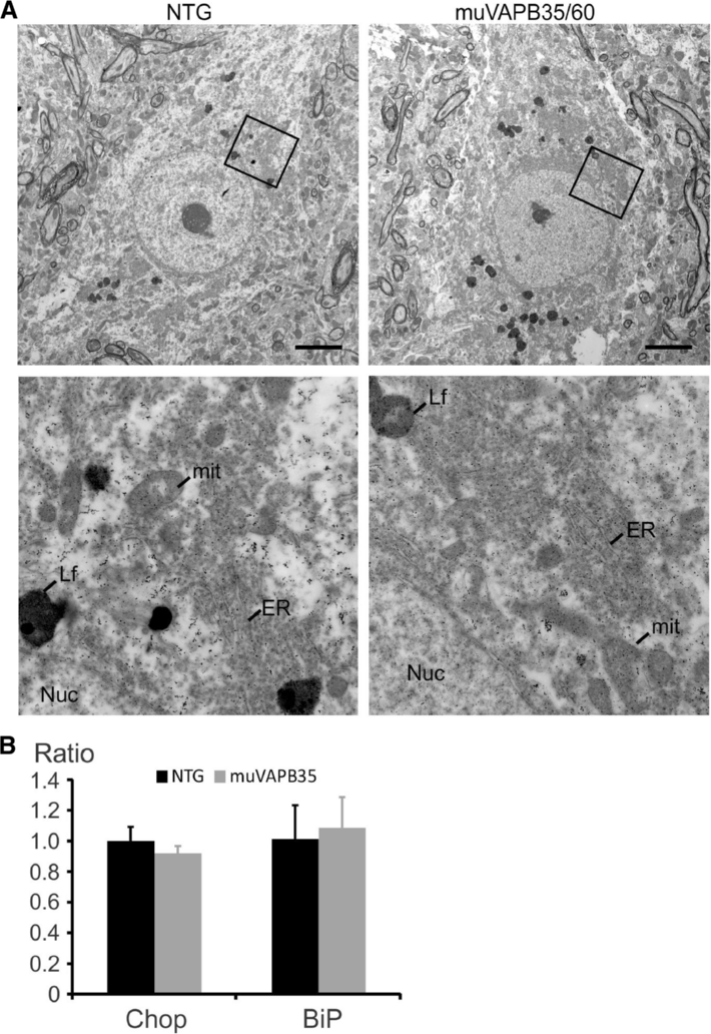
**muVAPB did not cause structural change for ER and mitochondria or induce UPR response.** (**A**) ER and mitochondrial morphology in the motor neurons from the lumbar spinal cords of muVAPB35/60 mice at 18-month old were not different from the NTG controls. (Abbreviations: Mit-mitochodria, Lf-lipofuscin, Nuc-nucleus, ER-endoplasmic reticulum.) Scale bars: 5 μm. (**B**) Relative ratios of Chop and BiP mRNA from the spinal cords of muVAPB35 mice at the age of 14 months as determined by RT-PCR and compared to the age-matched NTG mice. All values are represented by mean±SD. No significant difference was detected.

VAPB protein is normally located in the ER membrane [6]. Aggregation of mutant VAPB has been shown to be associated with ER stress [5,10,18,20-22]. In order to determine whether VAPB aggregates co-localize with ER proteins and have an adverse effect on ER integrity, we used PDI as a marker for ER structure and ER stress. We did not detect any change in the PDI staining pattern and PDI did not coaggregate with VAPB aggregates (Figure 6), suggesting the ER structure remains unaltered. We further compared the ER structure between spinal cord motor neurons of the muVAPB35/60 mice and NTG controls by electron microscopy (Figure 7A) and did not find any evidence of ER structural defect in the double mutant mice. Additionally, we examined mitochondrial morphology and could not find any notable differences between the double mutant mice and the age-matched NTG controls (Figure 7A). To determine whether there are functional changes, we investigated whether the UPR was activated. We quantified the expression levels of Chop (Ddit3) and BiP (Hspa5) in the muVAPB35 mice relative to those in the NTG mice by RT-PCR (Figure 7B). We did not detect any significant changes in the expression levels. Our results suggest that overexpression of mutant VAPB does not change the ER structure or induce the UPR.

**Figure 8:**
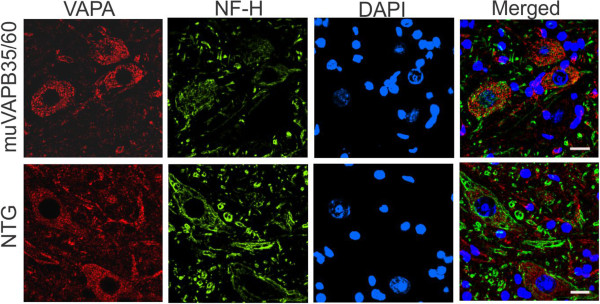
**muVAPB did not cause coaggregation of VAPA.** Sections from the lumbar spinal cords from NTG and muVAPB35/60 mice at 18-month old were stained for VAPA. NF-H was stained as a marker for neurons. The cell nuclei were shown by DAPI stain. Scale bars: 20 μm.

It has been shown that wild type VAPB and VAPA can be sequestered in the aggregates with muVAPB [12]. Because we did not have means to differentiate the transgenic VAPB and the endogenous wild type mouse VAPB, we chose to examine VAPA in the spinal cords from muVAPB35/60 double transgenic mice with VAPA antibody. However, we did not observe any VAPA aggregates, suggesting that VAPA was not sequestered in muVAPB aggregates (Figure 8).

### muVAPB aggregation did not affect disease progression in SOD1G93A mice

**Figure 9:**
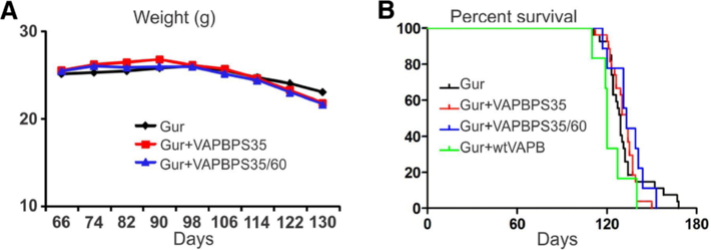
**Overexpression of neither wtVAPB nor muVAPB significantly impacted on the disease progression and survival of SOD1G93A mice.** (**A**) The effect of overexpression of muVAPB on the weight changes in SOD1G93A mice. The weight data of the male mice from SOD1G93A (n = 12), SOD1G93A and muVAPB35 double transgenic (n = 10), SOD1G93A and muVAPB35/60 triple transgenic mice (n = 10) were analyzed. (**B**) The survival of the male mice from SOD1G93A (n = 27), SOD1G93A and muVAPB35 double transgenic (n = 27), SOD1G93A and muVAPB35/60 triple transgenic (n = 27), and SOD1G93A and wtVAPB double transgenic mice (n = 6) were analyzed.

A previous study suggested that VAPB plays a role in the pathogenesis of ALS caused by Cu/Zn superoxide dismutase-1 (SOD1) mutations [12]. The study showed that the levels of VAPB were decreased in motor neurons of the mutant SOD1 mice, implicating the involvement of VAPB deficiency in the ALS disease progression. In order to investigate the possible effect of overexpressed VAPB on the disease progression of SOD1G93A transgenic mice, we crossed both wtVAPB and muVAPB transgenic mice with SOD1G93A ALS mice. We expected to observe an adverse effect of muVAPB overexpression on the disease course of SOD1G93A mice if there is a gain of toxic function or a dominant negative effect on the endogenous VAPB function. Additionally, if VAPB deficiency plays a significant role in the pathogenesis of ALS, we would observe a delayed onset and/or extended survival in the double transgenic SOD1G93A/wtVAPB mice. However, our results revealed that neither muVAPB nor wtVAPB overexpression altered the disease onset as determined by the peak body weight (Figure 9A) or the progression as determined by the lifespan (Figure 9B) in SOD1G93A mice. These results demonstrate that VAPB does not modulate the course of ALS disease.

**Figure 10:**
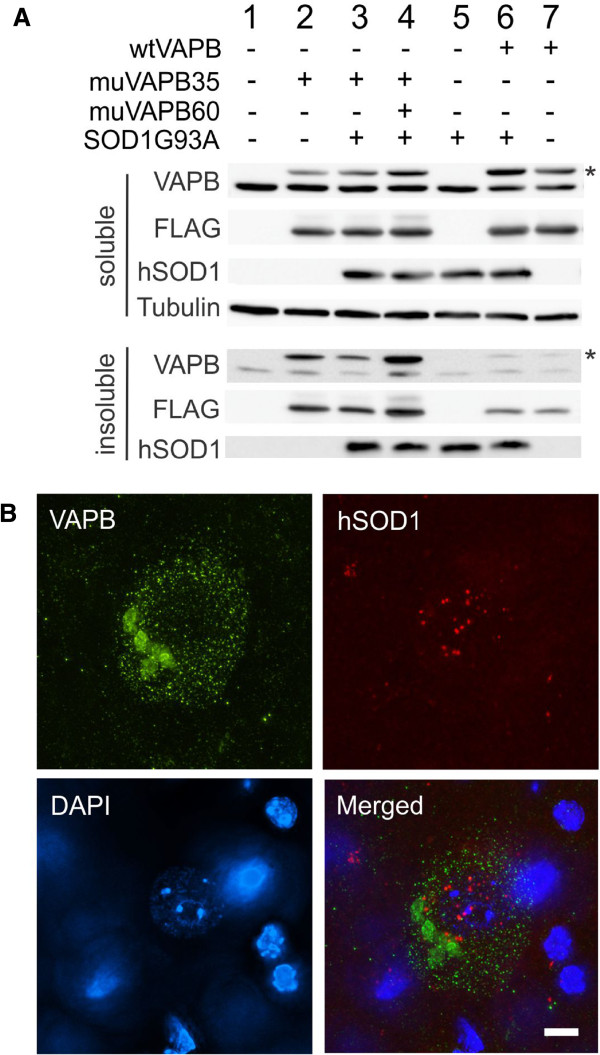
**VAPBP56S and SOD1G93A aggregates did not interact with each other.** (**A**). Mouse spinal cord lysates were centrifuged to separate the soluble and insoluble fractions. One hundred μg of both fractions were loaded on the gel and blotted for VAPB, FLAG, SOD1 and tubulin. The asterisks mark the 3XFLAG tagged VAPB, which is above the endogenous mouse VAPB. (**B**). muVAPB and SOD1G93A aggregate independent of each other in the same motor neuron in the spinal cord of the muVAPB35 and SOD1G93A double transgenic mice. The sections from the lumbar spinal cord were stained with VAPB and hSOD1 antibodies. The nuclei were shown by DAPI stain. Scale bar: 5 μm.

In some model systems, one mutant protein misfolding and aggregation can augment the misfolding and aggregation of another mutant protein [36]. To determine whether this type of interaction exist between VAPBP56S and SOD1G93A, we performed a sedimentation assay to compare the amount of aggregated protein in the single and double transgenic mice generated from the crosses between VAPB and SOD1G93A transgenic mice. The result showed that most of the endogenous mouse VAPB was soluble with only a small amount detected in the insoluble fraction (Figure 10A, lane 1). This pattern was not altered by the overexpression of muVAPB (lane 2), wtVAPB (lane 7), SOD1G93A (lane 5), both muVAPB and SOD1G93A (lanes 3 and 4) or both wtVAPB and SOD1G93A (lane 6). Noticeably, the fraction of insoluble muVAPB (lanes 2–4) was increased compared with the wtVAPB (lanes 6, 7). The increase of the insoluble muVAPB was particularly obvious in the muVAPB35/60 double transgenic mice (lane 4). But increased muVAPB did not significantly alter the insoluble amount of the endogenous VAPB (compare lanes 2–4 with lane 1) or the insoluble amount of mutant SOD1 (compare lanes 3 and 4 with lane 5). To determine whether muVAPB and SOD1G93A coaggregate, we carried out double immunofluorescence staining. We found that mutant VAPB and SOD1 formed independent aggregates in cells (Figure 10B). These results demonstrate that overexpression of muVAPB or wtVAPB does not impact on the insoluble fraction of the endogenous wild type VAPB and mutant SOD1, thus suggesting that there is no significant coaggregation of the endogenous wild type VAPB with the mutant VAPB and that the aggregation of muVAPB does not interact with the aggregation of the mutant SOD1.

## Discussion

Compared to the previous VAPB transgenic study [24], our results showed stronger and broader muVAPB aggregates, especially in motor neurons. Our muVAPB transgenic model with wide-spread aggregation formation could provide a useful tool to test the possible gain-of-toxic function due to protein misfolding and aggregation. Yet despite a systematic investigation with multiple analytic assays, we failed to find evidence for neurodegeneration or dysfunction in the transgenic mice. Although a two-fold overexpression of muVAPB may not have reached the toxic threshold, and therefore, may not be sufficient to trigger motor neuron degeneration, the failure of muVAPB to enhance mutant SOD1 toxicity diminishes the possibility of a harmful role of mutant VAPB in motor neuron degeneration. A further support for the innocuous nature of muVAPB is the absence of any pathological hallmarks, including structural changes of ER and mitochondria, evidence of UPR, reactive astrogliosis and microgliosis, redistribution of TDP-43, and activation of cellular protein quality control systems.

Our results contrast with some previous literature reports. Overexpression of muVAPB has been reported to alter ER morphology and modulate UPR *in vitro*[5,7,12,18]. Our data do not support these roles *in vivo*. In an analysis of a VAPBP56S transgenic mouse line, Tudor and colleagues reported a redistribution of TDP-43 from nucleus to the cytoplasm and the presence of ubiquitin- and p62-labeled structures in motor neurons [24]. We have not confirmed their observation in our muVAPB mice. The reason of this discrepancy is not clear. A simple explanation might be that the mutant VAPB was expressed at a higher level in their mice than in the mice used in this study. Consistent in both studies, however, is the observation that overexpression of muVAPB does not cause motor neuron degeneration phenotypes. Therefore, the results from both of these studies do not support the possibility that a gain of toxicity in mutant VAPB causes motor neuron degeneration.

Common in neurodegenerative diseases are cytoplasmic protein aggregates, including mutant or the wild type proteins of SOD1 [37,38], TDP-43 [39-41], and FUS/TLS [42]. However, it is not clear what roles these aggregates play in the pathogenesis of the disease. Indeed, it is controversial whether the aggregation is detrimental to cell survival. For example, neurodegeneration is still induced by ALS-related TDP-43 mutations in the absence of cytoplasmic TDP-43 aggregates, suggesting TDP-43 aggregation may not be required for pathogenesis [43,44]. Furthermore, inclusion body formation might protect neurons by reducing the levels of toxic soluble forms of mutants, as has been shown in mutant huntingtin [45]. It is possible that the neurodegeneration is not due to the presence of cytoplasmic aggregates, but by altered function of the mutant protein [46,47]. In human MND caused by VAPB mutations, the cellular mechanism leading to motor neuron degeneration and muscle atrophy is not known. Although robust VAPB aggregates were formed in the muVAPB transgenic mice, especially in the motor neurons, our results have revealed that motor neurons could tolerate the VAPB aggregates without degeneration, suggesting that VAPB protein aggregation may not play a significant role in the pathogenesis.

Whether VAPB aggregation has a role in human patients with VAPB mutations is not clear. In fact, it remains to be established that VAPB cytoplasmic aggregates exist in ALS8 patients. Motor neurons derived from iPS cells from ALS8 patients with the known VAPB-P56S mutation have decreased levels of VAPB without VAPB aggregation [48], which is consistent with the loss-of-function hypothesis. In this study, we detected no decrease in the endogenous VAPB level, nor did we attain evidence for involvement of VAPA or endogenous VAPB in the VAPB aggregates. This result does not support the possibility that the mutant VAPB exerts a dominant negative effect on the normal allele by coaggregation. Furthermore, in the presence of widespread VAPB aggregates, especially in the CNS, no neurodegeneration or motor dysfunction was observed, which does not support the toxic gain-of-function hypothesis. Therefore, future studies to determine whether a loss of function is the underlying pathogenic mechanism for the mutant VAPB is of critical importance.

Previous studies have shown that VAPB levels were reduced in sporadic ALS patients and SOD1G93A mice [12,26], suggesting that VAPB dysfunction may contribute to ALS in general. We tested this possibility by overexpression of either the mutant or the wild type VAPB in SOD1G93A mice. Our results demonstrate that neither the mutant nor the wild type VAPB had any impact on the course of the disease in the SOD1G93A mice. These results do not support a general role of VAPB in ALS cases that do not have VAPB mutations.

Since protein aggregation is common in many neurodegenerative diseases, it has been proposed that the protein aggregation weakens the capacity of cellular proteostasis, thus enhancing the cell vulnerability to secondary assault(s) from another misfolding-prone protein [35]. Our muVAPB/SOD1G93A double transgenic mice enabled us to test whether mutant SOD1 aggregation enhances mutant VAPB aggregation and vice versa in a mammalian system. Our data showed that VAPBP56S and SOD1G93A aggregate independently and do not enhance each other’s aggregation. Therefore, the synergistic protein misfolding and aggregation may not occur in all experimental systems. In addition, since VAPB aggregation did not cause motor neuron degeneration, and in particular, did not enhance mutant SOD1 toxicity, some protein aggregation may be innocuous and all protein aggregation does not inevitably lead to cellular toxicity.

## Conclusion

Overexpression of mutant but not wild type VAPB leads to robust VAPB-positive aggregates. However, motor neurons are capable of tolerating these aggregates without degeneration, thus suggesting that mutant VAPB does not cause ALS through toxicity associated with its aggregation. Furthermore, overexpression of either muVAPB or wtVAPB does not modulate protein aggregation and the course of the ALS in SOD1G93A mice, suggesting that VAPB does not play a significant role in ALS patients that do not have VAPB mutations. Importantly, mutant VAPB and mutant SOD1 aggregate independently and do not augment each other’s aggregation, thus suggesting that aggregation-prone proteins do not inevitably lead to deterioration of cellular proteostasis. Overall, our results do not support a gain of toxic function or a dominant negative effect as the underlying mechanism for muVAPB-induced neurodegeneration. Therefore, future experiments need to test the loss-of-function hypothesis.

## Methods

### Transgene constructs

Human VAPB (hVAPB) cDNA clone in the vector pCMV6-XL5 was purchased from OriGene. Proline at position-56 was changed to Serine by converting C to T at the 166th position using a site-directed mutagenesis kit (Qbiogene) with the PCR primers 5^′^-CCACGTAGGTACTGTGTGAGGTCCAACAGCGGAATCATCGAT-3^′^ and 5^′^-ATCGATGATTCCGCTGTTGGACCTCACACAGTACCTACGTGG-3^′^. The mutation was confirmed by sequencing both strands. Wild type hVAPB (hVAPBWT) and hVAPBP56S fragments were amplified by PCR with the primers flanked by EcoRI and KpnI sites and cloned in the plasmid vector p3XFLAG-CMV-7 (Sigma) with the same restriction enzyme sites to obtain two intermediate vectors (all enzymes were obtained from New England Biolabs unless otherwise indicated). The 3XFLAG tag was added to differentiate the transgene from the mouse endogenous VAPB gene. We used a previously described pCAG-EGFP/RFP-miRNAint plasmid [49] to construct the transgenic constructs (Figure 1A). The EGFP encoding fragment in the plasmid was cut out with Cla1 digestion, blunted by Klenow, and then cut by Sph1. The large fragment of the vector was gel-purified for subsequent cloning. The 3xFLAG-hVAPBWT and 3xFLAG-hVAPBP56S fragments were amplified by PCR from the intermediate vectors, blunted with T4 polymerase, and digested with SphI. The transgenic vectors pCAG-wtVAPB/RFP and pCAG-muVAPB/RFP were constructed by ligation of the large vector fragment and the 3xFLAG hVAPB WT or 3xFLAG hVAPB P56S fragment, respectively. All cloning procedures were verified by DNA sequencing.

### Generation and propagation of transgenic mice

The expression vectors, pCAG-wtVAPB/RFP and pCAG-muVAPB/RFP, were linearized by digestion with the restriction enzymes Ssp1 and Pci1 and subsequently purified as previously described [30]. The linearized DNA constructs were injected into C57BL/6 × SJL F2 hybrid mouse embryos by the UMassTransgenic Animal Modeling Core. Mouse tails of 2 mm in length were cut for PCR-based genotyping. Tail DNA samples were prepared by DirectPCR Lysis Reagent (Viagen Biotech) according to the provided instructions. Positive transgenic mice were identified by a 185 bp fragment within the open reading frame of the human VAPB gene produced by PCR using the primer pair 5^′^- AGTCTCTGAGTTCTTCTTTGG -3^′^ and 5^′^-CTTCCCAGTTGGGGCTA-3^′^. The PCR program was set as 4 minutes at 95°C, followed by 40 cycles of 95°C for 30 seconds, 51°C for 20 seconds, and 72°C for 30 seconds. The transgenic mice were maintained as heterozygotes by crossing with FVB/NJ mice. SOD1G93A (B6.Cg-Tg(SOD1*G93A)1Gur/J) transgenic mice [50] were obtained from Jackson Laboratory and crossed with FVB/NJ for ten or more generations. All mice were maintained at the University of Massachusetts Medical School animal facility according to the guidelines set forth by the Institutional Animal Care and Use Committee (IACUC) and all animal procedures were approved by the IACUC.

### Histological analyses

Mice under deep anesthesia were transcardially perfused with cold PBS followed by cold fixation buffer containing 4% paraformaldehyde in PBS. Tissues were dissected out and immersed in the same fixative overnight at 4°C. Tissues were treated in PBS containing 15% sucrose for 2–3 days at 4°C, and then frozen in OCT media (Sakura, Torrance, CA). Frozen sections at 10 μm thickness were cut by a cryostat and air-dried on slides at room temperature for 30 minutes. For immunofluorescence staining, slides were incubated in the blocking solution (5% normal goat serum and 0.2% Triton X-100 in PBS, pH7.4) for 30 min at RT, rinsed three times in PBS, and incubated with primary antibodies in the blocking solution overnight at 4°C. The slides were subsequently washed with PBS three times and incubated with secondary antibodies in the blocking solution for 2 hr at RT in dark. After washing, the slides were mounted with Vectashield mounting medium containing 4^′^,6^′^-diamidino-2-phenylindole (DAPI, Vector Laboratories). Images were taken with a Nikon Eclipse Ti microscope equipped with NIS elements software and further processed by the three-dimensional deconvolution program AutoQuant *X*2 (Media Cybernetics). The following antibodies were used: VAPB (ProteinTech Group), Iba1 (BioCare Medical), GFAP (Cell Signaling), TDP-43 (ProteinTech Group and Encor Biotechnology), NF-H (Covance), Ubiquitin (Millipore), PDI (Stressgen), and SOD1 (Biodesign). The secondary goat anti-rabbit, goat anti-mouse or donkey anti-sheep IgG (Invitrogen) conjugated by Alexa Fluor-488 or Alexa Fluor-555 were used.

For muscle histology, samples were obtained from fixed mice, embedded in paraffin blocks, sectioned and stained with Hematoxylin & Eosin (H&E).

For nerve and spinal cord plastic sections, the cervical and lumbar spinal cords and the L3 and L4 nerve roots were dissected from mice fixed with 4% paraformaldehyde and further fixed in 4% paraformaldehyde plus 2.5% glutaraldehyde in 0.1 M phosphate buffer, pH7.6 for 3 to 5 days. Plastic sections at 1 μm were processed and stained with toluidine blue. For electron microscopy, thin sections of ventral horn were cut from the Epon tissue blocks, stained with uranyl acetate and lead citrate, and proceeded for examination under a Philips CM-10 transmission electron microscope.

In all experiments, NTG mice were processed in parallel as negative controls. For ubiquitin and p62 staining, the end stage mutant SOD1 mice were used as positive controls.

### Western blotting

Mouse tissues were homogenized at a ratio of 100 mg/ml in homogenization solution (50 mM Tris HCl pH7.4, 1% SDS, 0.05% Triton X-100, 1 mM EGTA and protease inhibitor mixture) for 1 minute by using the Fast Prep-24 (MP Biomedicals). The resultant tissue lysates were heated at 95°C for 5 min followed by vortexing at low speed. After centrifugation at 13000 rpm for 5 min, the supernatants were stored at −80°C.

Protein samples (50 μg each) were resolved on a 10% SDS-polyacrylamide gel (Bio-Rad) for subsequent Western blotting. The membrane was probed with anti-FLAG (Stratagene,1:2000), anti-VAPB (ProteinTech Group, 1:1000), or anti-SOD1 antibody (Biodesign,1:10,000). The protein bands were visualized using the SuperSignal West Pico kit (Pierce). After detecting VAPB, the blot was stripped and reprobed with anti-tubulin primary antibody (Sigma, 1:10,000).

### Rotarod test

Before the actual test, mice were trained on an automated 4-lane rotarod unit (Columbus Instruments) for three days. The rotarod was set to an accelerating mode from 13 to 72 rpm over a period of 5 minutes. The animals were tested for three consecutive days and three times each day with a rest of 15 minutes between each trial. The length of time that each animal was able to stay on the rod was recorded. The best performance of each animal on each day was used to calculate the average performance for the three days.

### Grip strength test

A device with a force gauge (Chatillon, DFM2) connected with a stainless steel grid was used for the test. For forelimb grip strength measurement, the mouse was gently lowered over the top of the grid so that only its front paws can grip the grid. The mouse was pulled away from the grid until it released the grip. For combined forelimb and hind limb measurement, the mouse was lowered over the top of the grid so that both its front and hind paws can grip the grid. The mouse was pulled away horizontally and steadily until it released its grip. Each animal was measured three times and the peak grip strength value of the animal was recorded as its performance.

### RT-PCR

The RT-PCR was done as previously described [30]. Briefly, total RNA was extracted from mouse tissues by Trizol reagent (Invitrogen) followed by treatment with DNase I (Promega) and cDNA synthesis by using SuperScript III kit (Invitrogen). Quantitative PCR was performed by using Sybr Green mix (Qiagen). The primer pair 5^′^-TCCTGTCCTCAGATGAAATTGG-3^′^ and 5^′^-CCACTCTGTTTCCGTTTCCTA-3^′^ were designed for the mouse Chop gene (Ddit3) with a product size of 241 bp. The primer pair 5^′^-TTCAATGGCAAGGAGCCATC-3^′^ and 5^′^-TGATTATCGGAAGCCGTGGA-3^′^ were designed for the mouse BiP gene (Hspa5) with a product size of 239 bp. Mouse reference gene glyceraldehyde-3-phosphate dehydrogenase (Gapdh) was amplified with the primer pair 5^′^-CTGGAGAAACCTGCCAAGTA-3^′^ and 5^′^-TGTTGCTGTAGCCGTATTCA-3^′^, which produce a product of 223 bp. The PCR program was set as follows: 3 minutes at 95°C, followed by 40 cycles of 15 seconds at 95°C, 30 seconds at 58°C, and 20 seconds at 72°C. ΔCt values were calculated by subtracting the Ct values of Gapdh from those of Chop or BiP. ΔΔCt values were derived by subtracting the ΔCt values of the wild type from those of the transgenic mice, which were then used to calculate the levels of the mRNA in the transgenic mice relative to those in the controls. All the RT-PCR reactions were done by using the CFX96 Real-Time System (Bio-Rad).

### Sedimentation assay

Mice of different genotypes were generated from crossing wtVAPB transgenic or muVAPB35/60 double transgenic mice with SOD1G93A mice and were analyzed at the age of 95 to 100 days. Mouse tissues were mixed at a ratio of 1:10 with pre-chilled lysis buffer containing 50 mM Tris–HCl (pH 7.4), 1% Triton X-100, 1 mM EDTA, 150 mM KCl, and 1% multiple protease inhibitors (Sigma). Homogenization was done using the handheld polytron for 20 sec on ice. The lysates were centrifuged at 12,000 g for 5 min at 4°C. The supernatants were collected as the soluble fraction. The pellets were homogenized in the same buffer and centrifuged. The supernatants were discarded. The pellets were homogenized for 10 sec in the buffer containing 50 mM Tris HCl pH 7.4, 1% SDS, 0.05% Triton X-100, 1 mM EGTA and protease inhibitor mixture. One fifth of the original lysis buffer volume was used. Following the homogenization, the homogenate was vortexed vigorously for 10 sec. The resulting lysates were incubated at 95°C for 10 min and vortexed vigorously again for 10 sec. The samples were left at room temp to cool and then centrifuged at 13000 rpm for 5 min at room temperature. These supernatants were collected as the insoluble fraction. All the soluble and insoluble parts were used for Western blotting as stated above.

### Statistical analysis

Student’s *t*-test was used for statistical analysis.

## Abbreviations

VAPB: Vesicle-associated membrane protein-associated protein B; MND: Motor neuron disease; ALS: Amyotrophic lateral sclerosis; SMA: Spinal muscular atrophy; ER: Endoplasmic reticulum; UPR: Unfolded protein response; wt: Wild type; mu: Mutant; VAPA: Vesicle-associated membrane protein-associated protein A; P56S: Proline-to-serine substitution at position-56; MSP: Major sperm protein; T46I: Threonine to isoleucine at codon 46; fALS: Familial ALS; sALS: Sporadic ALS; NTG: Non-transgenic; CNS: Central nervous system; TDP43: TAR-DNA binding protein; FTD: Frontotemporal lobar degeneration; SOD1: Cu/Zn superoxide dismutase-1; H&E: Hematoxylin & Eosin; 3XFL: 3XFLAG; 3XpA: 3X poly A sequences.

## Competing interests

The authors declare that they have no competing interests.

## Authors’ contributions

MB and ZX designed the transgenic constructs. MB constructed and tested the vectors. LQ, MB, TQ, WT, HW, and BY performed experiments. LQ and ZX analyzed the data and wrote the manuscript. All authors have read and approved the final manuscript.

## References

[B1] Figlewicz DA Orrell RW The genetics of motor neuron diseases Amyotroph Lateral Scler Other Motor Neuron Disord 2003 4 225 231 10.1080/14660820310011287 14753656

[B2] Andersen PM Al-Chalabi A Clinical genetics of amyotrophic lateral sclerosis: what do we really know? Nat Rev Neurol 2011 7 603 615 10.1038/nrneurol.2011.150 21989245

[B3] Marques VD Barreira AA Davis MB Abou-Sleiman PM Silva WA Jr Zago MA Sobreira C Fazan V Marques W Jr Expanding the phenotypes of the Pro56Ser VAPB mutation: proximal SMA with dysautonomia Muscle Nerve 2006 34 731 739 10.1002/mus.20657 16967488

[B4] Nishimura AL Mitne-Neto M Silva HC Richieri-Costa A Middleton S Cascio D Kok F Oliveira JR Gillingwater T Webb J A mutation in the vesicle-trafficking protein VAPB causes late-onset spinal muscular atrophy and amyotrophic lateral sclerosis Am J Hum Genet 2004 75 822 831 10.1086/425287 15372378 PMC1182111

[B5] Chen HJ Anagnostou G Chai A Withers J Morris A Adhikaree J Pennetta G de Belleroche JS Characterization of the properties of a novel mutation in VAPB in familial amyotrophic lateral sclerosis J Biol Chem 2010 285 40266 40281 10.1074/jbc.M110.161398 20940299 PMC3001007

[B6] De Vos KJ Morotz GM Stoica R Tudor EL Lau KF Ackerley S Warley A Shaw CE Miller CC VAPB interacts with the mitochondrial protein PTPIP51 to regulate calcium homeostasis Hum Mol Genet 2012 21 1299 1311 10.1093/hmg/ddr559 22131369 PMC3284118

[B7] Fasana E Fossati M Ruggiano A Brambillasca S Hoogenraad CC Navone F Francolini M Borgese N A VAPB mutant linked to amyotrophic lateral sclerosis generates a novel form of organized smooth endoplasmic reticulum FASEB J 2010 24 1419 1430 10.1096/fj.09-147850 20008544

[B8] Kagiwada S Hosaka K Murata M Nikawa J Takatsuki A The Saccharomyces cerevisiae SCS2 gene product, a homolog of a synaptobrevin-associated protein, is an integral membrane protein of the endoplasmic reticulum and is required for inositol metabolism J Bacteriol 1998 180 1700 1708 9537365 10.1128/jb.180.7.1700-1708.1998 PMC107080

[B9] Kaiser SE Brickner JH Reilein AR Fenn TD Walter P Brunger AT Structural basis of FFAT motif-mediated ER targeting Structure 2005 13 1035 1045 10.1016/j.str.2005.04.010 16004875

[B10] Kanekura K Nishimoto I Aiso S Matsuoka M Characterization of amyotrophic lateral sclerosis-linked P56S mutation of vesicle-associated membrane protein-associated protein B (VAPB/ALS8) J Biol Chem 2006 281 30223 30233 10.1074/jbc.M605049200 16891305

[B11] Skehel PA Fabian-Fine R Kandel ER Mouse VAP33 is associated with the endoplasmic reticulum and microtubules Proc Natl Acad Sci U S A 2000 97 1101 1106 10.1073/pnas.97.3.1101 10655491 PMC15535

[B12] Teuling E Ahmed S Haasdijk E Demmers J Steinmetz MO Akhmanova A Jaarsma D Hoogenraad CC Motor neuron disease-associated mutant vesicle-associated membrane protein-associated protein (VAP) B recruits wild-type VAPs into endoplasmic reticulum-derived tubular aggregates J Neurosci 2007 27 9801 9815 10.1523/JNEUROSCI.2661-07.2007 17804640 PMC6672975

[B13] Skehel PA Martin KC Kandel ER Bartsch D A VAMP-binding protein from Aplysia required for neurotransmitter release Science 1995 269 1580 1583 10.1126/science.7667638 7667638

[B14] Amarilio R Ramachandran S Sabanay H Lev S Differential regulation of endoplasmic reticulum structure through VAP-Nir protein interaction J Biol Chem 2005 280 5934 5944 15545272 10.1074/jbc.M409566200

[B15] Soussan L Burakov D Daniels MP Toister-Achituv M Porat A Yarden Y Elazar Z ERG30, a VAP-33-related protein, functions in protein transport mediated by COPI vesicles J Cell Biol 1999 146 301 311 10.1083/jcb.146.2.301 10427086 PMC2156184

[B16] Pennetta G Hiesinger PR Fabian-Fine R Meinertzhagen IA Bellen HJ Drosophila VAP-33A directs bouton formation at neuromuscular junctions in a dosage-dependent manner Neuron 2002 35 291 306 10.1016/S0896-6273(02)00769-9 12160747

[B17] Morotz GM De Vos KJ Vagnoni A Ackerley S Shaw CE Miller CC Amyotrophic lateral sclerosis-associated mutant VAPBP56S perturbs calcium homeostasis to disrupt axonal transport of mitochondria Hum Mol Genet 2012 21 1979 1988 10.1093/hmg/dds011 22258555 PMC3315205

[B18] Tsuda H Han SM Yang Y Tong C Lin YQ Mohan K Haueter C Zoghbi A Harati Y Kwan J The amyotrophic lateral sclerosis 8 protein VAPB is cleaved, secreted, and acts as a ligand for Eph receptors Cell 2008 133 963 977 10.1016/j.cell.2008.04.039 18555774 PMC2494862

[B19] Han SM Tsuda H Yang Y Vibbert J Cottee P Lee SJ Winek J Haueter C Bellen HJ Miller MA Secreted VAPB/ALS8 major sperm protein domains modulate mitochondrial localization and morphology via growth cone guidance receptors Dev Cell 2012 22 348 362 10.1016/j.devcel.2011.12.009 22264801 PMC3298687

[B20] Gkogkas C Middleton S Kremer AM Wardrope C Hannah M Gillingwater TH Skehel P VAPB interacts with and modulates the activity of ATF6 Hum Mol Genet 2008 17 1517 1526 10.1093/hmg/ddn040 18263603

[B21] Langou K Moumen A Pellegrino C Aebischer J Medina I Aebischer P Raoul C AAV-mediated expression of wild-type and ALS-linked mutant VAPB selectively triggers death of motoneurons through a Ca2 + −dependent ER-associated pathway J Neurochem 2010 114 795 809 10.1111/j.1471-4159.2010.06806.x 20477942

[B22] Suzuki H Kanekura K Levine TP Kohno K Olkkonen VM Aiso S Matsuoka M ALS-linked P56S-VAPB, an aggregated loss-of-function mutant of VAPB, predisposes motor neurons to ER stress-related death by inducing aggregation of co-expressed wild-type VAPB J Neurochem 2009 108 973 985 10.1111/j.1471-4159.2008.05857.x 19183264

[B23] Kim S Leal SS Ben Halevy D Gomes CM Lev S Structural requirements for VAP-B oligomerization and their implication in amyotrophic lateral sclerosis-associated VAP-B(P56S) neurotoxicity J Biol Chem 2010 285 13839 13849 10.1074/jbc.M109.097345 20207736 PMC2859547

[B24] Tudor EL Galtrey CM Perkinton MS Lau KF De Vos KJ Mitchell JC Ackerley S Hortobagyi T Vamos E Leigh PN Amyotrophic lateral sclerosis mutant vesicle-associated membrane protein-associated protein-B transgenic mice develop TAR-DNA-binding protein-43 pathology Neuroscience 2010 167 774 785 10.1016/j.neuroscience.2010.02.035 20188146

[B25] Ratnaparkhi A Lawless GM Schweizer FE Golshani P Jackson GR A Drosophila model of ALS: human ALS-associated mutation in VAP33A suggests a dominant negative mechanism PLoS One 2008 3 e2334 10.1371/journal.pone.0002334 18523548 PMC2390852

[B26] Anagnostou G Akbar MT Paul P Angelinetta C Steiner TJ de Belleroche J Vesicle associated membrane protein B (VAPB) is decreased in ALS spinal cord Neurobiol Aging 2011 31 969 985 18701194 10.1016/j.neurobiolaging.2008.07.005

[B27] Chai A Withers J Koh YH Parry K Bao H Zhang B Budnik V Pennetta G hVAPB, the causative gene of a heterogeneous group of motor neuron diseases in humans, is functionally interchangeable with its Drosophila homologue DVAP-33A at the neuromuscular junction Hum Mol Genet 2008 17 266 280 17947296 10.1093/hmg/ddm303 PMC3516386

[B28] Boillee S Yamanaka K Lobsiger CS Copeland NG Jenkins NA Kassiotis G Kollias G Cleveland DW Onset and progression in inherited ALS determined by motor neurons and microglia Science 2006 312 1389 1392 10.1126/science.1123511 16741123

[B29] Yamanaka K Chun SJ Boillee S Fujimori-Tonou N Yamashita H Gutmann DH Takahashi R Misawa H Cleveland DW Astrocytes as determinants of disease progression in inherited amyotrophic lateral sclerosis Nat Neurosci 2008 11 251 253 10.1038/nn2047 18246065 PMC3137510

[B30] Qiu L Rivera-Perez JA Xu Z A Non-specific effect associated with conditional transgene expression based on Cre-loxP strategy in mice PLoS One 2011 6 e18778 10.1371/journal.pone.0018778 21572998 PMC3091857

[B31] DeJesus-Hernandez M Mackenzie IR Boeve BF Boxer AL Baker M Rutherford NJ Nicholson AM Finch NA Flynn H Adamson J Expanded GGGGCC hexanucleotide repeat in noncoding region of C9ORF72 causes chromosome 9p-linked FTD and ALS Neuron 2011 72 245 256 10.1016/j.neuron.2011.09.011 21944778 PMC3202986

[B32] Arai T Hasegawa M Akiyama H Ikeda K Nonaka T Mori H Mann D Tsuchiya K Yoshida M Hashizume Y Oda T TDP-43 is a component of ubiquitin-positive tau-negative inclusions in frontotemporal lobar degeneration and amyotrophic lateral sclerosis Biochem Biophys Res Commun 2006 351 602 611 10.1016/j.bbrc.2006.10.093 17084815

[B33] Neumann M Sampathu DM Kwong LK Truax AC Micsenyi MC Chou TT Bruce J Schuck T Grossman M Clark CM Ubiquitinated TDP-43 in frontotemporal lobar degeneration and amyotrophic lateral sclerosis Science 2006 314 130 133 10.1126/science.1134108 17023659

[B34] Mackenzie IR Rademakers R Neumann M TDP-43 and FUS in amyotrophic lateral sclerosis and frontotemporal dementia Lancet Neurol 2010 9 995 1007 10.1016/S1474-4422(10)70195-2 20864052

[B35] Balch WE Morimoto RI Dillin A Kelly JW Adapting proteostasis for disease intervention Science 2008 319 916 919 10.1126/science.1141448 18276881

[B36] Gidalevitz T Ben-Zvi A Ho KH Brignull HR Morimoto RI Progressive disruption of cellular protein folding in models of polyglutamine diseases Science 2006 311 1471 1474 10.1126/science.1124514 16469881

[B37] Bergemalm D Forsberg K Srivastava V Graffmo KS Andersen PM Brannstrom T Wingsle G Marklund SL Superoxide dismutase-1 and other proteins in inclusions from transgenic amyotrophic lateral sclerosis model mice J Neurochem 2010 114 408 418 10.1111/j.1471-4159.2010.06753.x 20412382

[B38] Bruijn LI Becher MW Lee MK Anderson KL Jenkins NA Copeland NG Sisodia SS Rothstein JD Borchelt DR Price DL Cleveland DW ALS-linked SOD1 mutant G85R mediates damage to astrocytes and promotes rapidly progressive disease with SOD1-containing inclusions Neuron 1997 18 327 338 10.1016/S0896-6273(00)80272-X 9052802

[B39] Gitcho MA Baloh RH Chakraverty S Mayo K Norton JB Levitch D Hatanpaa KJ 3rd White CL Bigio EH Caselli R TDP-43 A315T mutation in familial motor neuron disease Ann Neurol 2008 63 535 538 10.1002/ana.21344 18288693 PMC2747362

[B40] Kabashi E Valdmanis PN Dion P Spiegelman D McConkey BJ Vande Velde C Bouchard JP Lacomblez L Pochigaeva K Salachas F TARDBP mutations in individuals with sporadic and familial amyotrophic lateral sclerosis Nat Genet 2008 40 572 574 10.1038/ng.132 18372902

[B41] Sreedharan J Blair IP Tripathi VB Hu X Vance C Rogelj B Ackerley S Durnall JC Williams KL Buratti E TDP-43 mutations in familial and sporadic amyotrophic lateral sclerosis Science 2008 319 1668 1672 10.1126/science.1154584 18309045 PMC7116650

[B42] Vance C Rogelj B Hortobagyi T De Vos KJ Nishimura AL Sreedharan J Hu X Smith B Ruddy D Wright P Mutations in FUS, an RNA processing protein, cause familial amyotrophic lateral sclerosis type 6 Science 2009 323 1208 1211 10.1126/science.1165942 19251628 PMC4516382

[B43] Barmada SJ Skibinski G Korb E Rao EJ Wu JY Finkbeiner S Cytoplasmic mislocalization of TDP-43 is toxic to neurons and enhanced by a mutation associated with familial amyotrophic lateral sclerosis J Neurosci 2010 30 639 649 10.1523/JNEUROSCI.4988-09.2010 20071528 PMC2821110

[B44] Wegorzewska I Bell S Cairns NJ Miller TM Baloh RH TDP-43 mutant transgenic mice develop features of ALS and frontotemporal lobar degeneration Proc Natl Acad Sci U S A 2009 106 18809 18814 10.1073/pnas.0908767106 19833869 PMC2762420

[B45] Arrasate M Mitra S Schweitzer ES Segal MR Finkbeiner S Inclusion body formation reduces levels of mutant huntingtin and the risk of neuronal death Nature 2004 431 805 810 10.1038/nature02998 15483602

[B46] Xu Z Does a loss of TDP-43 function cause neurodegeneration? Mol Neurodegener 2012 7 27 10.1186/1750-1326-7-27 22697423 PMC3419078

[B47] Lee JH Yu WH Kumar A Lee S Mohan PS Peterhoff CM Wolfe DM Martinez-Vicente M Massey AC Sovak G Lysosomal proteolysis and autophagy require presenilin 1 and are disrupted by Alzheimer-related PS1 mutations Cell 2010 141 1146 1158 10.1016/j.cell.2010.05.008 20541250 PMC3647462

[B48] Mitne-Neto M Machado-Costa M Marchetto MC Bengtson MH Joazeiro CA Tsuda H Bellen HJ Silva HC Oliveira AS Lazar M Downregulation of VAPB expression in motor neurons derived from induced pluripotent stem cells of ALS8 patients Hum Mol Genet 2010 20 3642 3652 21685205 10.1093/hmg/ddr284 PMC3159551

[B49] Qiu L Wang H Xia X Zhou H Xu Z A construct with fluorescent indicators for conditional expression of miRNA BMC Biotechnol 2008 8 77 10.1186/1472-6750-8-77 18840295 PMC2569932

[B50] Gurney ME Pu H Chiu AY Dal Canto MC Polchow CY Alexander DD Caliendo J Hentati A Kwon YW Deng HX Motor neuron degeneration in mice that express a human Cu,Zn superoxide dismutase mutation Science 1994 264 1772 1775 10.1126/science.8209258 8209258

